# Hyperoxia evokes pericyte-mediated capillary constriction

**DOI:** 10.1177/0271678X221111598

**Published:** 2022-07-03

**Authors:** Chanawee Hirunpattarasilp, Anna Barkaway, Harvey Davis, Thomas Pfeiffer, Huma Sethi, David Attwell

**Affiliations:** 1 Department of Neuroscience, Physiology and Pharmacology, University College London, Gower Street, London, UK; 2 Princess Srisavangavadhana College of Medicine, Chulabhorn Royal Academy, Bangkok, Thailand; 3 Division of Neurosurgery, UCL Queen Square Institute of Neurology, Queen Square, London, UK

**Keywords:** Hyperoxia, pericyte, 20-HETE, reactive oxygen species, cerebral blood flow

## Abstract

Oxygen supplementation is regularly prescribed to patients to treat or prevent hypoxia. However, excess oxygenation can lead to reduced cerebral blood flow (CBF) in healthy subjects and worsen the neurological outcome of critically ill patients. Most studies on the vascular effects of hyperoxia focus on arteries but there is no research on the effects on cerebral capillary pericytes, which are major regulators of CBF. Here, we used bright-field imaging of cerebral capillaries and modeling of CBF to show that hyperoxia (95% superfused O_2_) led to an increase in intracellular calcium level in pericytes and a significant capillary constriction, sufficient to cause an estimated 25% decrease in CBF. Although hyperoxia is reported to cause vascular smooth muscle cell contraction via generation of reactive oxygen species (ROS), endothelin-1 and 20-HETE, we found that increased cytosolic and mitochondrial ROS levels and endothelin release were not involved in the pericyte-mediated capillary constriction. However, a 20-HETE synthesis blocker greatly reduced the hyperoxia-evoked capillary constriction. Our findings establish pericytes as regulators of CBF in hyperoxia and 20-HETE synthesis as an oxygen sensor in CBF regulation. The results also provide a mechanism by which clinically administered oxygen can lead to a worse neurological outcome.

## Introduction

Inhaled oxygen supplementation is often given clinically, with the aim of treating or preventing tissue hypoxia.^1^ However, excessive oxygen administration leads to hyperoxia, a state in which tissue or organs are exposed to a supraphysiological level of oxygen (in brain tissue, more than 50 mmHg or 65 µM).^2–6^ In a study of patients with traumatic head injuries, increasing the fraction of inspired oxygen (FiO_2_) from around 0.3 to 1.0 (i.e. from breathing 30% to breathing 100% O_2_) raised the brain tissue oxygen tension (PbtO_2_) from approximately 30 mmHg to 150 mmHg in one example trace.^7^ Moreover, an average PbtO_2_ of 220 mmHg was found in 26 traumatic brain injury patients treated with hyperbaric oxygenation (FiO_2_ 1.0 at 1.5 × atmospheric pressure for 1 hour).^8^ Hyperoxia is very prevalent in clinical practice, but frequently left uncorrected.^9^

However, besides showing no benefit for patients with hypoxic brain conditions such as acute stroke,^10^ hyperoxia is associated with worsened neurological outcomes in preclinical studies^11^ and in patients with cardiac arrest, subarachnoid haemorrhage and ischaemic stroke.^12–15^ Individuals can also develop life-threatening central nervous system (CNS) oxygen toxicity when exposed to a hyperbaric hyperoxic gas mixture.^16^

One of the mechanisms by which hyperoxia affects the brain is through a reduction of cerebral blood flow (CBF).^17^ A decrease in CBF of 10–30% in (normobaric) hyperoxic healthy subjects was found using techniques such as angiography and MRI,^17–19^ and constrictions of the internal carotid arteries and vertebral arteries have been detected.^19^ During hyperbaric oxygenation, cerebral blood flow was also reduced in animals^20, 21^ and more hypo-perfused areas were found in professional divers.^22^ Although breathing in more oxygen increases blood oxygen content, some studies have shown that the reduction in CBF might compromise oxygen delivery to the brain,^17^ thus disrupting the energy supply to neurons.

Many mechanisms of hyperoxia-induced constriction in arteries and arterioles have been proposed, including the following.

- *Increased production of reactive oxygen species (ROS)*. Application of superoxide dismutase in rats^23^ or ascorbic acid in human patients,^19^ both of which have anti-oxidant properties, was able to inhibit the hyperoxia-induced decrease of CBF. This is consistent with a role for ROS in generating hyperoxic vasoconstriction. Moreover, an increased ROS level in mouse brains was found in hyperoxic conditions.^24^ In hyperoxia, ROS might originate from NADPH oxidase (NOX) activity, especially of NOX2 and NOX4, or from mitochondria, because their ROS-generating activities are increased at a high oxygen level.^25–28^ One mechanism by which ROS cause vasoconstriction is through scavenging the vasodilator nitric oxide (NO).^23, 24^
- *Increased endothelin-1 (ET) release.* A high level of oxygen induces secretion of the vasoconstrictor ET from endothelial cells^29, 30^ and increased cerebral release of big-ET, a precursor of ET,^31^ was observed after experimental hyperoxia in neurosurgical patients.^32^ In addition, blocking ET receptors partly reduced the effect of hyperoxia on retinal blood flow in newborn piglets.^33^
- *Generation of the vasoconstrictor 20-hydroxyeicosatetraenoic acid (20-HETE).* Hyperoxia has been shown to increase 20-HETE generation in the peripheral circulation^34^ and in the retina.^33, 35^ The 20-HETE synthesizing enzyme has an EC_50_ for binding O_2_ of 55 µM, i.e. in the physiological range of brain O_2_ levels.^36^

Most studies of hyperoxic vasoconstriction have focused on the contraction of vascular smooth muscle cells on arteries and arterioles. However, most of the vascular resistance in the cerebral microcirculation is in capillaries^37–41^ (but see^42^) and the regulation of blood flow in capillaries is at least partly mediated by contraction and relaxation of pericytes.^38, 43^ Since there might be different mechanisms underlying capillary and arteriolar regulation of CBF,^44^ understanding how hyperoxia affects pericyte contractility would provide insight into how hyperoxia decreases CBF, which might explain why hyperoxia is related to poorer neurological outcome in some patients. Furthermore, such studies will also raise awareness of the negative effect on the brain of administering excessive oxygen in clinical practice, and of the fact that brain slices are often oxygenated with an unphysiologically high oxygen level in electrophysiological studies.

## Materials and methods

### Extracellular solution

Experiments with live brain slices from rodents and humans used aCSF containing (in mM) 124 NaCl, 2.5 KCl, 1 MgCl_2_, 2 CaCl_2_, 1 NaH_2_PO_4_, 26 NaHCO_3_, 10 D-glucose, and 0.1 Na-ascorbate, bubbled with gas containing 5% CO_2_ (pH 7.4).

### Rodent brain slices

All animal procedures were performed in accordance with EU and UK regulations (the UK Animals (Scientific Procedures) Act 1986 and subsequent modifications) and approval by the UCL Animal Welfare and Ethical Review Body, and are described in accordance with the ARRIVE guidelines.^45^ The experiments used P19–P22 Sprague-Dawley rats and P57–P58 NG2-CreERT2 × PC::G5-tdT mice. NG2-CreERT2 × PC::G5-tdT mice were obtained from an in-house cross of NG2-DsRedBAC mice (JAX stock # 008241)^46^ and PC::G5-tdT mice (JAX stock # 024477)^47^ – in pericytes, smooth muscle cells and oligodendrocyte precursor cells they express a red fluorescent protein, tdTomato and, when induced with tamoxifen, produce the genetically encoded calcium indicator GCaMP5G.

Animals were sacrificed humanely by cervical dislocation followed by decapitation. The animal’s head was immediately immersed in ice-cold recovery solution containing (in mM) 92 NaCl, 2.5 KCl, 1 MgCl_2_, 2 CaCl_2_, 1.2 NaH_2_PO_4_, 30 NaHCO_3_, 20 HEPES, 25 D-glucose, 5 Na-ascorbate, 3 Na-pyruvate, 1 kynurenic acid (to block glutamate receptors to prevent neuronal damage by glutamate released during the slicing procedure), with NaOH to bring the pH to 7.35, oxygenated with 95% O_2_/5% CO_2_. The brain was extracted and sliced into 300 µm thick sections in ice-cold slicing solution containing (in mM) 93 NMDG, 2.5 KCl, 10 MgCl_2_, 0.5 CaCl_2_, 1.2 NaH_2_PO_4_, 30 NaHCO_3_, 20 HEPES, 25 D-glucose, 5 Na-ascorbate, 3 Na-pyruvate, 1 kynurenic acid, with HCl to bring the pH to 7.35, bubbled with 95% O_2_/5% CO_2_. The slices were incubated in the same solution at 35°C. After 20 minutes, the brain slices were transferred to the recovery solution at room temperature, for at least 10 minutes before use and then in most experiments superfused with normal aCSF solution containing 20% O_2_ (for the time needed to find a suitable vessel to image (∼10 mins) plus a 5 minute baseline period) before examining the effects of hyperoxia. When drugs were applied they were present for a further 15 mins after this baseline period before the switch to 95% O_2_.

### Human brain slices

Human brain slices were employed to extend to humans the most important results obtained from rodents. Ethical approval from the National Health Service (REC number 12/NW/0568) and informed consent from all patients were received. Only anonymised details of age, sex and disease condition of the patients were passed on from the surgeon. All other patient data were kept confidential. All procedures involving human tissue were in accordance with the Human Tissue Act (2014).

Apparently normal cortical brain tissue, which is removed for access in neurosurgical operations for brain tumours, and which would otherwise be discarded, was placed in ice-cold slicing solution pre-oxygenated with 95% O_2_/5% CO_2_. The tissue was transported to the laboratory in less than 15 min. Cerebral cortical slices were prepared as for rodent slices.

### Imaging of capillaries and arterioles

Brain slices were transferred to a bright-field microscope and perfused, while suitable vessels for experiments were identified, with aCSF oxygenated with 20% O_2_, 5% CO_2_, and 75% N_2_ at 33–36°C with a flow rate of 2.5–4 ml/min. 20% O_2_ has been shown to produce an approximately physiological [O_2_] in the slice (10–40 µM) at the depths where we normally image^48^ and was used to mimic normoxic conditions. Solution bubbled with 95% O_2_ and 5% CO_2_, as is commonly used in electrophysiological studies, was used to mimic hyperoxic conditions because this produces an unphysiologically high [O_2_] in the slice of 285–345 µM,^49^ i.e. a PO_2_ of 220–265 mmHg, which is similar to the value for hyperbaric hyperoxic patients.^7, 8^ Healthy capillaries (<10 µm diameter without rings of arteriolar smooth muscle around them) were identified at 20–50 µm depths from the slice surface in the grey matter of the cortex. Regions which had a candidate pericyte (identified by a bump-on-a-log morphology) and for which at least 30 µm length along the vessel was in focus, were chosen and imaged every 30 s by a CCD camera with a pixel size of 200 nm. Vessels were not pre-constricted except where this is explicitly stated.

Measurement of vessel internal diameters in the resulting images were performed by manually placing a measurement line perpendicular to the vessel at the pericyte location using Metamorph Software (Molecular Devices). There was no difference between the hyperoxia-evoked vasoconstriction of 14 male vessels and that of 6 female vessels (p = 0.57).

### Calculation of effect of vascular diameter changes on blood flow

This calculation is based on the method previously described.^50^ The resistance to flow ( R ) was calculated from Poiseuille’s law (which assumes laminar flow of a pure liquid and thus ignores the presence of cells in the blood), in which

$$R=\frac{kL}{{r_{1}}^{4}}$$

where k is a constant, L is the length of the vessel from a pericyte soma to midway between two pericytes and ${\;r}_{1\;}$ is the vessel radius which is initially assumed to be equal along the whole vessel. When pericytes constrict or dilate capillaries, the capillary radius will then decrease or increase from a value of ${\;r}_{1}$ at the midpoint between pericytes to a value of ${\;r}_{2}$ at the pericyte soma. For this situation, assuming that the radius changes linearly with the distance, the resistance can be calculated as:

$$R=\frac{kL({r_{1}}^{2}+r_{1}r_{2}+{r_{2}}^{2})}{{3r_{1}}^{3}{r_{2}}^{3}}$$

Relative to the resistance ( $R_{1}$ ) for when the vessel radius is assumed to be spatially uniform at $r_{1}$ , the factor by which the resistance is changed is (equation (1))

$$\frac{R}{R_{1}}=\frac{\left[1+\frac{r_{1}}{r_{2}}+{\left(\frac{r_{1}}{r_{2}}\right)}^{2}\right]\times \frac{r_{1}}{r_{2}}}{3}$$

For example, normally, when a pericyte is relaxed, the capillary radius at the pericyte soma is ∼1.1 times that between pericytes $r_{1}$ ,^50^ for which equation (1) predicts that the resistance of the capillary segment will be *R_relaxed_/R_1_* = 83% of the value it would have if the diameter were not 10% dilated at the soma. If a vasoconstrictor is applied which produces a constriction of X % at the pericyte soma, then the ratio of the new radius at the soma ( $r_{2}$ ) to $r_{1}$ will be (equation (2))

$$\frac{r_{2}}{r_{1}}=\left(\frac{100-X}{100}\right)\times 1.1$$

By inserting the result from equation (2) into equation (1), it is possible to calculate the factor by which the resistance is changed (*R_contracted_/R_1_*) relative to the whole capillary having diameter $r_{1}$ . Then, by taking the ratio of this new constricted resistance to the value (relative to the whole capillary having diameter $r_{1})\;$ when the pericyte is relaxed (in the absence of the constrictor: 0.83, see above), the effect of the constrictor on the resistance of the capillary segment can be calculated.

Because capillaries confer 57% of the total vascular resistance within the brain parenchyma^39^ and flow is inversely correlated with vascular resistance, the new flow will be changed by a factor of (equation (3))

$$\frac{\left(43\%+57\%\right)}{\left[43\%+(\mathrm{Rcontr}a\mathrm{cted}/\mathrm{Rrelaxed})\times 57\%\right]}$$

where we assume that all pericytes constrict capillaries by the same amount and we only take into account changes in the resistance of capillaries, without any changes being assumed to occur in arterioles and venules.

### Calcium imaging

Calcium imaging was employed to probe mechanisms underlying changes of pericyte contractile tone. Brain slices from NG2-CreERT2 × PC::G5-tdT mice were transferred to a two-photon microscope and perfused with aCSF gassed with 20% O_2_, 5% CO_2_ and 75% N_2_ at 33–36°C with a flow rate of 3–4 ml/min, while a suitable vessel was found. Pericytes were identified by their tdTomato signal and morphology resembling a bump-on-a-log using a water immersion 20 × objective. A region of interest, which was at least 20 µm in depth and had at least 3 pericytes in the image stack, was identified. Laser power and digital gain were adjusted to obtain a suitable intensity. Pericytes were excited with a 940 nm excitation wavelength laser and signals from tdTomato (labelling pericyte outline) and GCaMP5G (to measure the calcium signal in pericytes) were collected simultaneously. The image focus was readjusted after every five image stacks were taken (∼2.5 min).

An image sequence of maximum intensity Z-projections of the image stacks was created and aligned using the ImageJ Fiji program function StackReg. The pericyte soma was delineated and its calcium intensity profile across time was measured and plotted. The averages of five intensity measurements around the times of interest (normally the peak of any Ca^2+^ transient), were used for further statistical analysis.

### ROS imaging

For experiments to measure cytosolic superoxide with dihydroethidium (DHE), cortical slices made as described above from P19–P23 Sprague-Dawley rats were incubated in aCSF with DHE 8 µM at 35°C and oxygenated with either 95% O_2_ (and 5% CO_2_ – the hyperoxic condition) or 20% O_2_ (and 5% CO_2_, 75% N_2_ – the normoxic condition) for 1 h. The DHE was then washed off with ice-cold aCSF, and slices were then immediately imaged live under a confocal microscope. Image stacks were taken randomly in the cortex with a 20x objective and the mean fluorescence of the plane with maximum intensity was measured.

To measure mitochondrial superoxide, we incubated rat brain slices with aCSF in hyperoxic or normoxic conditions for 1 h and then loaded the cells with MitoSOX 5 µM for 15 min. The slices were fixed with 4% PFA at room temperature for 1 h, washed three times in PBS for 10 min and imaged under a confocal microscope the next day. Image stacks were taken at random. For each image stack, the mean fluorescence of the image plane with the maximum intensity was measured.

### Statistical analysis

All statistical analyses were performed using Prism 7.00 (GraphPad). Data are presented as mean ± s.d. Normality of data was examined by the Shapiro-Wilk normality test. Equality of variance between two normally distributed data sets was examined using the F test. Two-tailed Student’s t-tests and t-tests with Welch’s correction were used when the variance was equal or unequal respectively. Comparison of data that failed the normality test was made using a Mann-Whitney test. To correct for multiple comparisons within a figure panel, a procedure similar to the Holm-Bonferroni method was used (i.e. for N comparisons, the smallest P-value is multiplied by N, the second smallest P-value by N-1, etc.). Corrected p-values of less than 0.05 were considered significant. For experiments with pharmacological inhibition of hyperoxic vascular changes, vessel diameters were normalised to the values in the last 10 images of the vessels when the inhibitors alone were applied before application of hyperoxia. Drug and control experiments were interleaved in a non-blinded manner. Source data for figures are available as a Supplementary File.

## Results

### Hyperoxia induces capillary constriction in rat and human cortical slices

**Figure 1.**
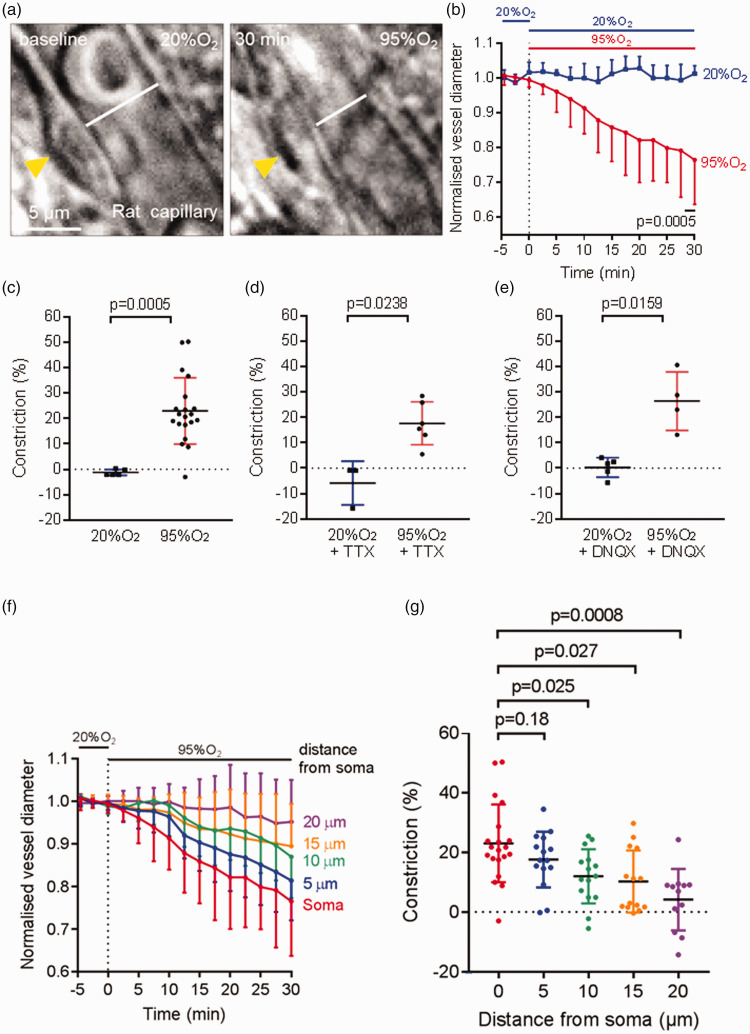
Hyperoxia-evoked capillary constriction in rat cerebral cortical tissue. (a) Representative bright-field images of a rat cortical capillary, with a pericyte indicated by the yellow arrow, in the normoxic condition (left) and 30 min after application of hyperoxic aCSF oxygenated with 95% O_2_ (right), showing capillary constriction evoked by hyperoxia. The white lines in both images display the vessel diameter. (b) Time course of capillary diameter on applying aCSF oxygenated with 95% O_2_ (n = 20) or aCSF oxygenated with 20% O_2_ (also present in the baseline period, n = 5) for 30 min. Dotted line indicates the time when application of hyperoxic solution started. (c) Constriction in (b) at t = 28–30 min, showing a significant capillary constriction in the presence of hyperoxia in rat cortex. (d–e) As in (c) but in the presence of 2 µM TTX (d, n = 3 in 20% and n = 6 in 95% O_2_) or 10 µM DNQX (e, n = 5 in 20% and n = 4 in 95% O_2_). (f) Mean constriction as a function of distance along the capillary from 20 pericyte somata, on changing from 20% to 95% O_2_ in the superfusate and (g) Mean constriction from (f) after 30 mins of 95% O_2_ (dots show individual measurements).

To investigate the effect of hyperoxia on the diameters of 1st–4th branch order capillaries (measured from a penetrating arteriole), we perfused aCSF oxygenated with 95% O_2_ (the hyperoxic condition) onto rat cortical brain slices for 30 min, after a 5 min baseline period of superfusing aCSF with 20% O_2_ (the normoxic condition). Hyperoxia caused a capillary constriction of 23.0 ± 13.0% (n = 20) while continuing oxygenation with 20% O_2_ caused a vasoconstriction of −1.2 ± 1.2% (n = 5, significantly different, p = 0.0005, Figure 1(a) to (c)). This hyperoxia-induced vasoconstriction was also observed in the presence of 2 µM TTX to block action potentials (Figure 1(d)) or 10 µM DNQX to block AMPA receptors and the glycine-binding site of NMDA receptors (Figure 1(e)). In the presence of these drugs the 24% constriction evoked in 95% [O_2_] (relative to in 20% [O_2_] was not significantly affected (23% in TTX, 26% in DNQX, p = 0.36 and 0.48 respectively compared to the data in Figure 1(c)).

The hyperoxia-evoked constriction was focussed at pericyte somata (Figure 1(f) and (g)). This is expected, given that most of the circumferential processes of pericytes (which have the appropriate orientation to alter capillary diameter) are located within 10 µm of the pericyte soma: see Figs. S2 and S3 of ref.^49^).

Previous work has suggested that the response of contractile mural cells to vasoactive agents depends on the initial level of tone in the cells, with constrictions evoked in relaxed vessels even being converted to dilations in preconstricted vessels.^51^ However, pre-constricting capillaries at pericytes by superfusing 2 µM noradrenaline did not convert the hyperoxia-evoked constriction into a dilation. In aCSF bubbled with 20% O_2_, noradrenaline constricted vessels at pericyte somata by 28.1 ± 7.9% (n = 4) after 45 mins. If this was repeated but bubbling with 95% O_2_ for the last 30 mins, the constriction was 44.1 ± 27.5% (n = 6). Although this 16% increase in constriction did not reach statistical significance (p = 0.23), we can rule out the possibility that hyperoxia evokes a dilation in pre-constricted capillaries. Furthermore, there was actually a weak positive correlation between the constriction evoked in each capillary after 15 mins noradrenaline superfusion and the subsequent increase in constriction when the superfusate oxygen level was switched from 20% to 95% for 30 mins (p = 0.12 comparing the slope of the resulting linear regression line with zero), implying that preconstriction with noradrenaline did not tend to reduce the hyperoxia-evoked constriction.

**Figure 2.**
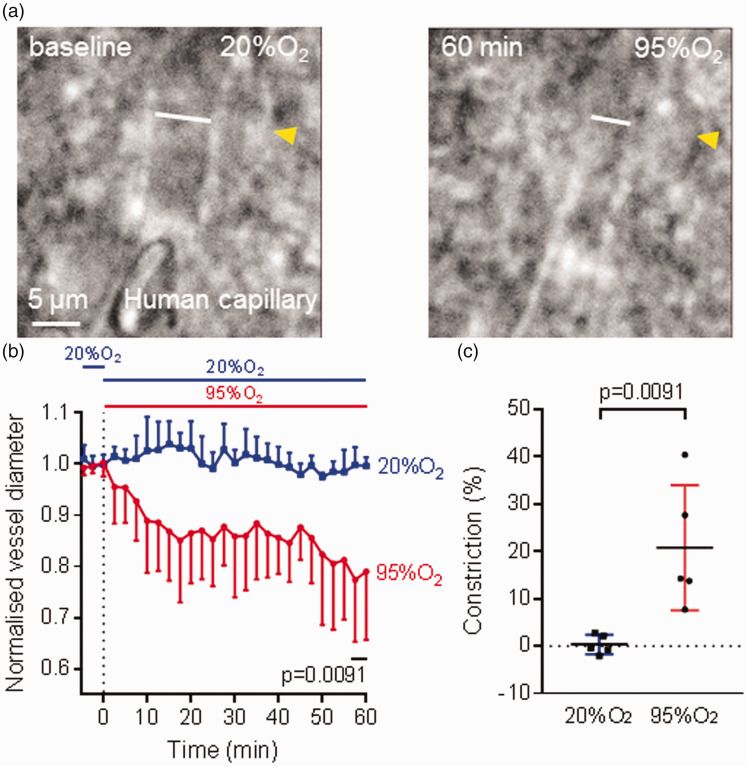
Hyperoxia-evoked capillary constriction in human cerebral cortical tissue. (a) Bright-field images of a human cortical capillary with a pericyte (yellow arrow) in the normoxic condition (left) and 60 min after application of hyperoxic aCSF oxygenated with 95% O_2_ (right), showing capillary constriction. The white lines represent vessel diameter. (b) Capillary constriction at pericyte locations in human cortical tissue was found after application of hyperoxic aCSF (95% O_2_, n = 5) but not with normoxic aCSF (20% O_2_, also present in the baseline period, n = 5). Dotted line indicates the time when application of hyperoxia started. (c) Constriction in (b) at t = 58-60 min, showing that hyperoxia evoked a significant capillary constriction in human cerebral cortex.

Hyperoxic capillary constriction was also found in human brain tissue obtained from neurosurgical operations. In human cortical slices, a capillary constriction of 20.8 ± 13.2% (n = 5) was found after the slices were perfused with hyperoxic solution for 1 h compared to a vasoconstriction of 0.4 ± 2.1% (n = 5, p = 0.0091, Figure 2(a) to (c)) found with continued perfusion of normoxic solution (20% O_2_).

We used a Poiseuille’s law analysis (see Materials and Methods) to estimate the likely flow change that this constriction would produce. As explained in the Materials and Methods section, when pericytes are relaxed the capillary radius at capillary positions between pericytes (*r_1_*) is less than that at the pericyte soma (*r_2_*) with (*r_2_*/*r_1_*)∼1.1. This ratio is reduced by hyperoxia by a multiplicative factor of 0.792 (from the 20.8% constriction given above). Inserting these two values of (*r_2_*/*r_1_*) into equation (1), and the resulting resistances into equation (3), predicts that the decrease of blood flow occurring in hyperoxia (assuming that only pericytes, and not arterioles or venules, are affected by the hyperoxia) will be ∼25%.

These experiments show that hyperoxia causes pericyte-mediated capillary constriction and thus may evoke a cerebral blood flow deficit in both rats and humans.

### Hyperoxia increases pericyte [Ca^2+^]_i_

Pericyte contractility is positively correlated with intracellular calcium concentration^41, 52^ because calcium is needed for calmodulin-induced activation of myosin light chain kinase, resulting in phosphorylation of the myosin light chain that interacts with α-smooth muscle actin to cause contraction.^53^

**Figure 3.**
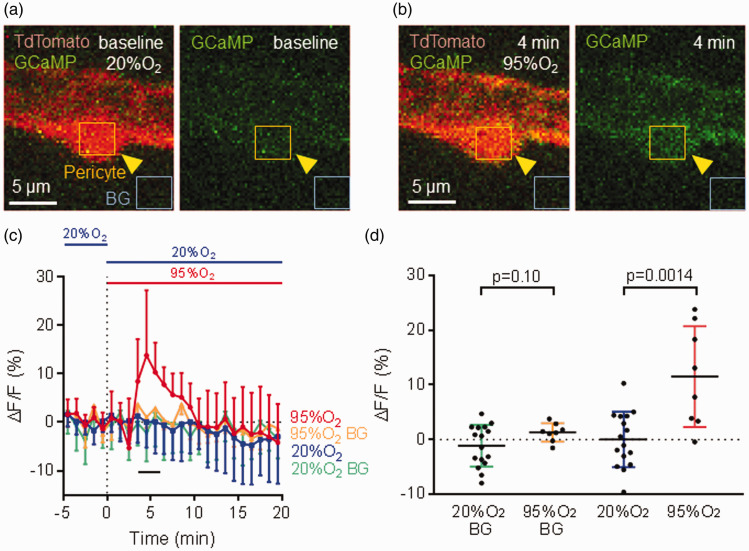
Hyperoxia-evoked [Ca^2+^]_i_ increase in mouse cerebral cortex pericytes. (a and b) Fluorescent images of a cortical pericyte (yellow arrows) from NG2-CreERT2 x PC::G5-tdT mice, which express TdTomato (red) and the calcium indicator GCaMP5G (green) in pericytes, in the normoxic condition (a) and 4 min after application of hyperoxic solution (b). Superimposed squares show regions of interest (ROIs) over the pericyte and over a background (BG) part of the image. There was a rise in GCaMP5G fluorescence in the pericyte (but not in the background) after superfusing hyperoxic aCSF, indicating that [Ca^2+^]_i_ is increased. (c) Time course of the increase of GCaMP5G fluorescence (ΔF/F) on application of aCSF oxygenated with 95% O_2_ (n = 8) or aCSF oxygenated with 20% O_2_ (also used for the baseline period, n = 16) for 20 min, showing a peak increase of [Ca^2+^]_i_ at 4–6 min followed by a decrease in the maintained presence of high [O_2_]. Background ROIs showed no significant change. (d) ΔF/F in (C) averaged over t = 4-6 min, showing a significant increase in GCaMP5G fluorescence in pericytes after application of aCSF oxygenated with 95% O_2_, with no significant change in background ROIs.

Brain slices from NG2-CreERT2 × PC::G5-tdT mice were used to quantify calcium signals in pericytes. Hyperoxia evoked an increase in GCaMP fluorescence in pericytes (Figure 3(a) and (b)) with the largest percentage increase (ΔF/F), averaged over the period from 4–6 min in hyperoxic solution, of 11.5 ± 9.2% (n = 8). This was significantly different (p = 0.0014) from the ΔF/F of 0.0 ± 5.1% (n = 16) for capillaries perfused with normoxic solution (Figure 3(c) and (d); note that Figure 3(c) shows the exact value at each time point but statistical analysis was performed using the average intensity from 5 frames). Hyperoxia evoked no significant fluorescence change in background areas away from pericytes. Thus, hyperoxia raises the intracellular calcium concentration in pericytes, which presumably generates the capillary constriction shown above.

### Reactive oxygen species are generated in hyperoxic conditions but do not mediate the hyperoxic vasoconstriction

Since hyperoxia has been shown to increase cellular ROS production,^25^ we performed live cell staining with DHE to visualise cytosolic superoxide production and with MitoSOX to visualise mitochondrial superoxide production, in order to identify the source of ROS that are generated in hyperoxic conditions. MitoSOX is a mitochondrially-targeted superoxide indicator that accumulates in the mitochondrial matrix.^54^

**Figure 4.**
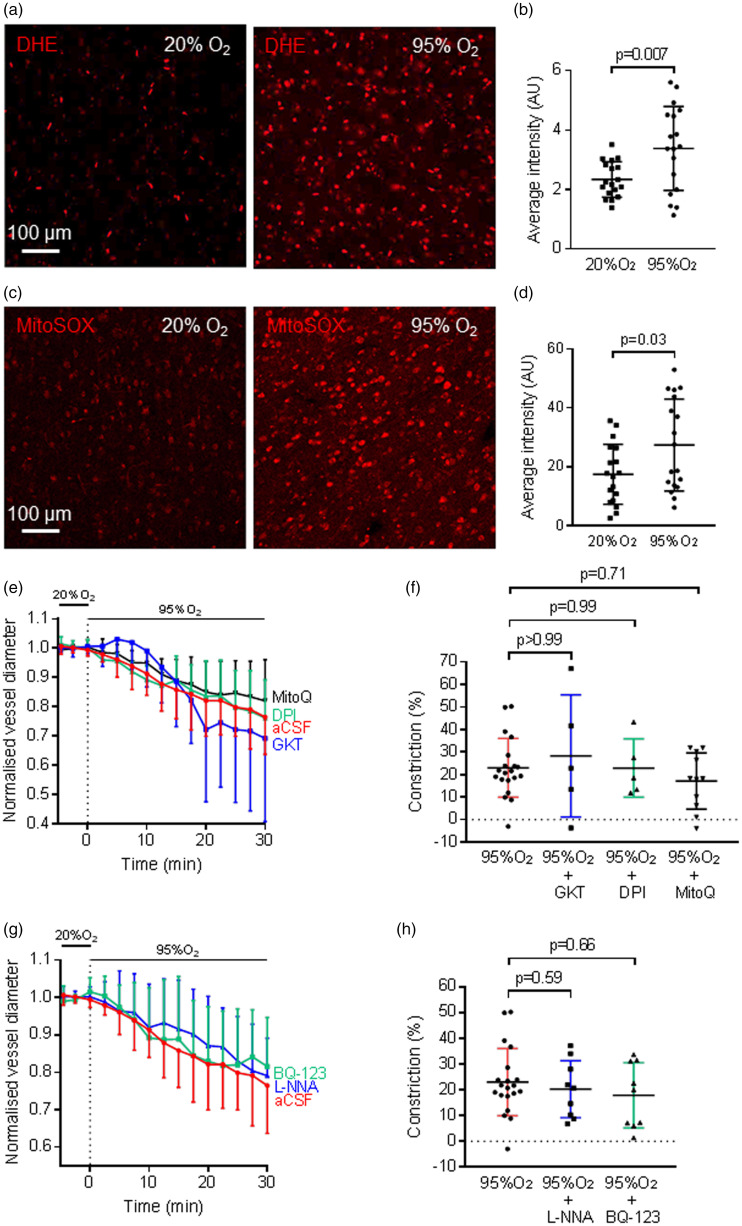
Hyperoxia induces cytosolic and mitochondrial ROS production but ROS and endothelin do not mediate hyperoxic capillary constriction. (a) Dihydroethidium (DHE) staining of rat cortical slices incubated in aCSF with 20% O_2_ (left) and with 95% O_2_ (right), to visualise cytosolic superoxide production. Cytosolic reactive oxygen species (ROS) production was increased when the slices were made hyperoxic. (b) Average intensity of DHE fluorescence from rat cortical slices incubated in aCSF oxygenated with 20% O_2_ or with 95% O_2_, showing a significantly higher level of fluorescence in hyperoxic slices (n = 18 for both conditions). (c) MitoSOXstaining for mitochondrial ROS production in rat brain slices in normoxic and hyperoxic conditions, showing an increase in mitochondrial ROS production when the slices were in hyperoxic solution. (d) Average intensity of MitoSOX staining of rat cortical slices incubated in aCSF oxygenated with 20% O_2_ (normoxic) or incubated in aCSF oxygenated with 95% O_2_ (hyperoxic), showing that mitochondrial ROS production was enhanced in hyperoxia (n = 18 for both conditions). (e) Hyperoxic aCSF (n = 20, red trace) induced capillary constriction occurred with a similar time course in the presence of the NOX4 inhibitor GKT137831 (GKT, 0.45 µM, n = 5, blue trace), the nonspecific NOX blocker DPI (10 µM, n = 5, green trace) or the mitochondrially-targeted antioxidant MitoQ (500 nM, n = 11, black trace). Inhibitors were applied for 15 min before application of 95% O_2_ (dotted line) and diameters were normalised to the pre-hyperoxia baseline. (f) Constriction in (e) averaged over t = 28–30 min, showing that hyperoxic vasoconstriction was not inhibited by GKT137831, DPI or MitoQ. (g) Time course of capillary diameter on applying aCSF oxygenated with 95% O_2_ (n = 20, red trace) alone, or in the presence of the nitric oxide synthase inhibitor N_ω_-nitro-L-arginine (L-NNA, 100 µM, n = 9, blue trace) or the endothelin-1 type A receptor (ET_A_) inhibitor BQ-123 (1 µM, n = 9, green trace). Diameters were normalised to the pre-hyperoxic phase (before the dotted line). (h) Constriction in (g) averaged over t = 28-30 min, showing that the hyperoxia-evoked capillary constriction was not significantly blocked by L-NNA or BQ-123.

When compared to application of aCSF oxygenated with 20% O_2_, application of aCSF oxygenated with 95% O_2_ significantly increased the mean fluorescence of DHE (n = 18 in each condition, p = 0.0068, Figure 4(a) and (b)) and of MitoSOX (n = 18 in each condition, p = 0.030, Figure 4(c) and (d)), suggesting that both cytosolic and mitochondrial ROS production in the cerebral cortex are increased in hyperoxia. ROS can be generated enzymatically, primarily by NADPH oxidase (NOX), and non-enzymatically through the mitochondrial electron transport chain.^28^ Therefore, we examined whether NOXs, especially NOX4, which is the main NOX in the vasculature^55^ and produces more ROS when the concentration of oxygen is higher,^26^ and mitochondria were involved in hyperoxic capillary constriction.

We applied the specific NOX4 inhibitor GKT137831 (0.45 µM)^50^ or the nonspecific NOX blocker diphenyleneiodonium (DPI, 10 µM)^50^ for 15 min before application of hyperoxia and measured capillary diameter changes near pericytes in rat cortical slices. These agents alone (in normoxia) did not evoke significant capillary diameter changes after 15 min (GKT137831: −2.5 ± 2.0%, n = 5, p = 0.13; DPI: 5.0 ± 3.4%, n = 5, p = 0.063). GKT137831 failed to stop the hyperoxia-induced vasoconstriction: in its presence, 30 min of hyperoxia evoked a capillary constriction of 28.3 ± 27.1% (n = 5, p > 0.99 compared to hyperoxic capillary constriction without any blocker, Figure 4(e) and (f)). Similarly, DPI (10 µM) did not prevent hyperoxic vasoconstriction, with hyperoxia evoking a constriction of 22.9 ± 12.9% (n = 5, p = 0.99 compared to no DPI, Figure 4(e) and (f)).

Investigation of the involvement of mitochondrial ROS in hyperoxic vasoconstriction in rat brain slices was performed by perfusing a mitochondrially-targeted antioxidant,^56, 57^ MitoQ (500 nM), for 15 min before superimposing 95% O_2_. MitoQ (500 nM) alone (in normoxia) did not evoke a significant capillary diameter change after 15 min (0.0 ± 3.5%, n = 11, p = 0.97). Hyperoxia for 30 min in the presence of MitoQ then evoked a constriction of 17.2 ± 12.5% (n = 11, p = 0.71 compared to in the absence of the blocker, Figure 4(e) and (f)).

These experiments suggest that NOXs and mitochondria are not involved in the hyperoxic capillary constriction. Since studies have shown that hyperoxia can constrict cerebral arteries through the nitric oxide (NO)-scavenging effect of superoxide,^23^ we tested whether the nitric oxide synthase inhibitor N_ω_-nitro-L-arginine (L-NNA) could inhibit the hyperoxia-evoked capillary constriction. In its presence, a capillary constriction of 20.3 ± 11.1% (100 µM, n = 9, p = 0.59, Figure 4(g) and (h)) was found after application of 95% O2 for 30 min. This implies that depletion of NO is not the cause of this vasoconstriction. In normoxia, L-NNA (100 µM) alone did not evoke a significant capillary diameter change after 15 min (0.9 ± 10.5%, n = 9, p = 0.73).

### Hyperoxia does not evoke capillary constriction through endothelin signalling

To investigate whether ET is involved in hyperoxic vasoconstriction, we perfused an inhibitor of vasoconstricting endothelin-1 type A (ET_A_) receptors (BQ-123, 1 µM) for 15 min onto rat cortical slices before increasing the oxygen level to 95% O_2_. Hyperoxia in the presence of BQ-123 evoked a vasoconstriction of 17.8 ± 12.7% (n = 9, p = 0.66 compared to the constriction evoked by hyperoxia without any blocker, Figure 4(g) and (h)), indicating that ET does not contribute to hyperoxia-evoked capillary vasoconstriction. BQ-123 (1 µM) alone did not evoke a significant capillary diameter change after 15 min (−0.3 ± 9.7%, n = 9, p = 0.43) in normoxia.

#### 20-HETE is involved in hyperoxic capillary constriction

**Figure 5.**
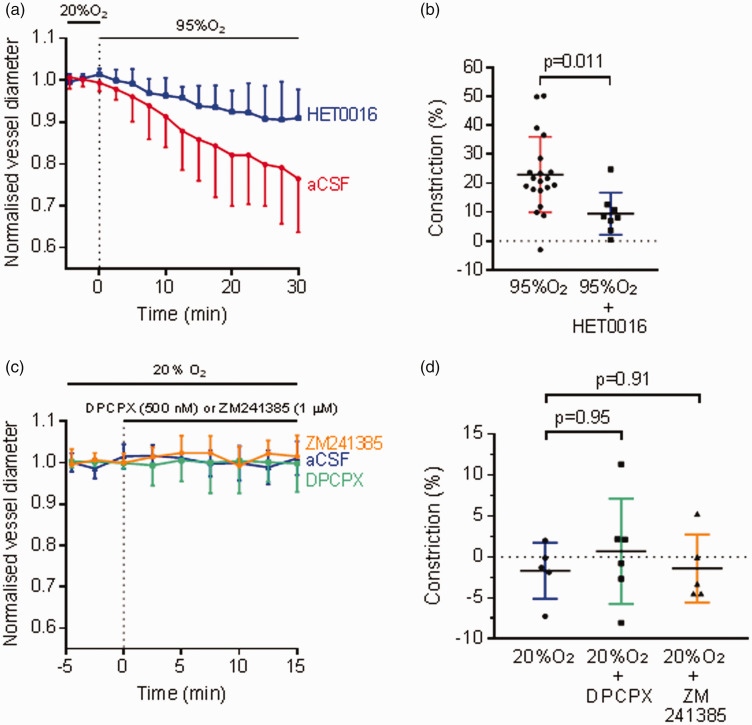
Hyperoxia-induced vasoconstriction is partly mediated by 20-HETE. (a) Hyperoxic vasoconstriction in rat cortical slices (n = 20) was partly inhibited by application of HET0016 (100 nM, n = 8), an inhibitor of the synthesis of 20-hydroxyeicosatetraenoic acid (20-HETE) which did not affect the baseline capillary diameter. Diameters were normalised to the pre-hyperoxic phase (before the dotted line). (b) Constriction in (a) averaged over t = 28-30 min, showing that application of HET0016 partially blocked hyperoxia-evoked capillary constriction in rat cortical slices. (c) Time course of capillary diameter on applying aCSF oxygenated with 20% O_2_ (n = 5) alone, or in the presence of the adenosine A1 receptor inhibitor 8-cyclopentyl-1,3-dipropylxanthine (DPCPX, 500 nM, n = 6, green trace) or the adenosine A2a receptor inhibitor ZM241385 (1 μM, n = 5, orange trace). Dotted line indicates the time when application of drugs started. (d) Constriction in (c) averaged over t = 13-15 min, showing that the application of DPCPX and ZM241385 did not have a significant effect on capillary diameter.

Increased production of the vasoconstricting arachidonic acid derivative 20-hydroxyeicosatetraenoic acid (20-HETE) has been shown to be one of the mechanisms underlying hyperoxic vasoconstriction in the peripheral circulation^34^ and in the retina.^35^ However, the involvement of 20-HETE generation in hyperoxia-evoked pericyte-mediated cerebral capillary contraction has not been studied. In rat brain slices, the 20-HETE synthesis inhibitor HET0016 (100 nM) inhibited the hyperoxic vasoconstriction evoked by 30 min of 95% O_2_ by 59%, reducing the constriction to 9.5 ± 7.3% (n = 8) from a constriction of 23.0 ± 13.0% (n = 20, p = 0.011, Figure 5(a) and (b)) without the blocker. Application of HET0016 (100 nM) alone for 15 min did not evoke a significant vasoconstriction (1.1 ± 3.8%, n = 8, p = 0.31). Thus, hyperoxia evokes pericyte contraction partly via 20-HETE production.

### Hyperoxic vasoconstriction is not caused by lowering the extracellular level of adenosine, ATP or PGE_2_

When brain slices were oxygenated with 20% O_2_, the centre of the slices could become hypoxic,^48^ leading to an artifactual generation of adenosine,^58^ a well-known vasodilator. We explored whether this adenosine production, which could be suppressed by hyperoxia,^49^ contributed to the larger capillary diameter seen in slices gassed with 20% O_2_ and hence to its reduction in hyperoxia. We inhibited adenosine’s A1 and A2a receptors, which cause pericyte hyperpolarisation^59^ and increase capillary blood flow,^60^ in brain slices oxygenated with 20% O_2_. Blocking A1 receptors with 8-cyclopentyl-1,3-dipropylxanthine (DPCPX, 500 nM) or blocking A2a receptors with ZM241285 (1 µM) did not cause a significant diameter change after 15 min (DPCPX: 0.7 ± 6.4%, n = 6, p = 0.95 compared to normoxia without drug; ZM241385: −1.4 ± 4.1%, n = 5, p = 0.91, Figure 5(c) and (d)). This suggests that any artifactual increase in adenosine level does not affect capillary diameter in normoxic conditions, and so a suppression of this process by hyperoxia is not responsible for causing hyperoxic vasoconstriction.

Similarly, although electrically-evoked neuronal activity evokes ATP release that evokes capillary dilation via astrocyte P2X_1_ receptors,^44^ in the absence of electrical stimulation we found that the P2X_1_ blocker NF449 (0.1 µM) neither affected the capillary diameter in 20% O_2_ (increased by 2% in 9 capillaries after 15 mins, not significant, p = 0.98), nor affected the subsequent constriction evoked by 95% O_2_ (29.5 ± 9.4% in 5 capillaries after 30 mins of 95% O_2_, not significantly different to that in Figure 1(b), p = 0.62). Thus, the possibility that hyperoxia evokes a constriction by suppressing ATP release can be excluded.

It has also been suggested^61^ that in 20% O_2_ accumulation of lactate leads to a higher extracellular concentration of prostaglandin E_2_, which dilates capillaries at pericytes,^44^ implying that the lower lactate level occurring in 95% O_2_ would evoke constriction. However, applying L-161,982 (1 µM) to block the EP4 receptor that mediates^44^ PGE_2_-evoked dilation had no significant effect on the baseline capillary diameter in 20% O_2_ (a 3% constriction in 10 capillaries,^44^ p = 0.3), implying that this mechanism does not contribute significantly to the hyperoxia-induced constriction, at least in our brain slice experiments.

## Discussion

These experiments provide two key results for understanding cerebral capillary diameter responses to hyperoxia: (i) a high level of oxygen causes pericyte contraction, resulting in smaller capillary diameters and thus reduced cerebral blood flow; and (ii) hyperoxia-evoked capillary constriction is mediated partly via 20-HETE.

Perfusing hyperoxic solution caused pericyte contraction and capillary constriction in both rat and human brain cortical slices, with a predicted *in vivo* blood flow reduction of ∼25% from human tissue experiments. This prediction is comparable to the results of studies in patients, which showed a cerebral blood flow deficit of 10–30% after inhalation of hyperoxic air in normobaric or hyperbaric conditions.^17–21^

Supporting a role for pericytes in hyperoxic vasoconstriction, we found a rapid rise in pericyte intracellular calcium concentration in response to hyperoxic solution. As calcium is needed for the activation of the myosin light chain kinase that promotes binding of actin and myosin,^53^ pericyte calcium concentration controls pericyte contraction.^41, 52^ Surprisingly, the [Ca^2+^]_i_ rise showed a slow decay while contraction continued to increase (compare Figures 3(c) and 1(b)), however this Ca^2+^ transient with a slow decay pattern in pericytes during hyperoxia is similar to that seen in pericyte Ca^2+^ when applying other vasoconstrictors.^62, 63^ A long-lasting contraction even after [Ca^2+^]_i_ returns towards its normal level is also observed after ET-1 application to pericytes.^62, 64^ This is suggested to occur as a result of Ca^2+^-sensitising pathways, such as through Rho kinase or PKC,^64^ both of which are stimulated by 20-HETE (see below).^65–67^ Rho kinase, in particular, acts by inhibiting myosin light chain phosphatase, thus increasing the phosphorylation of myosin light chain and the contraction evoked by a given rise of [Ca^2+^]_i_.

We tested several mechanisms of hyperoxic vasoconstriction proposed in studies of cerebral arteries, to see whether they were relevant to hyperoxia-induced pericyte contraction, as follows.

### Production of ROS

We found that the production of both cytosolic and mitochondrial ROS in cerebral cortex were increased in hyperoxia (Figure 4(a) to (d)). This is consistent with other studies showing that hyperoxia increases ROS generation in the cytosol of cells in the caudal solitary complex in rats^68^ and in the mitochondria of rat brain cortex.^69^ Surprisingly, however, blocking NOXs (including NOX4, which has been shown to increase activity in high oxygen^26^) or reducing mitochondrial ROS levels with MitoQ, was not sufficient to suppress hyperoxic capillary constriction. It appears, therefore, that hyperoxia-evoked capillary constriction is not mediated through ROS.

Studies on cerebral arteries have shown that hyperoxia promotes the production of superoxide, which binds to NO to create peroxynitrite^70, 71^ and thus attenuates NO-dependent vasodilation.^23, 24^ This mechanism does not contribute to pericyte-mediated hyperoxic constriction of capillaries because blocking NO production with L-NNA did not prevent the constriction (Figure 4(g) to (h)). This may reflect the fact that, unlike in arterioles, NO is not involved in mediating cortical capillary dilation.^44^

From these experiments, it can be concluded that there is an increased overall ROS production in both the cytosol and mitochondria in hyperoxia, but ROS apparently do not contribute to generating hyperoxia-induced vasoconstriction.

### Release of endothelin (ET)

Although several studies have shown that ET is produced in hyperoxic conditions,^29, 30^ blocking vasoconstricting endothelin signalling with BQ-123 did not prevent hyperoxic capillary vasoconstriction (Figure 4(g) to (h)). Thus ET signalling does not contribute to the pericyte-mediated vasoconstriction.

### Production of 20-HETE

Increased 20-HETE production via a reaction catalysed by CYP4A occurs in hyperoxia.^33–35^ This reflects the fact that this reaction in rats has a Michaelis constant (K_m_) of ∼55 µM for O_2_ at 37°C, so increasing the oxygen level superfusing brain slices from 20% O_2_ (giving an [O_2_] in the tissue of ∼40 µM) to 95% O_2_ (giving an [O_2_] in the tissue of ∼125 µM) would accelerate this reaction.^6, 36^ 20-HETE causes vasoconstriction in the cerebral circulation^72, 73^ by inducing contraction of vascular smooth muscle cells^74, 75^ and pericytes.^76^ This occurs by activation of L-type Ca^2+^ channels via inhibition of large conductance Ca^2+^-activated K^+^ channels and PKC signalling,^65, 66^ activation of Rho kinase^67^ and stimulation of NOX to increase superoxide production in vascular smooth muscle.^74^ Thus, production of 20-HETE is well poised to be an underlying mechanism in hyperoxic vasoconstriction.

Indeed, we found that HET0016, a 20-HETE synthesis blocker, reduced hyperoxia-induced capillary constriction (Figure 5(a) and (b)). Oxygen-induced 20-HETE generation might also contribute to the increased ROS production found in hyperoxia (Figure 4(a) and (b)), because 20-HETE has been shown to increase ROS production from NADPH oxidases^74^ and mitochondria.^77^

HET0016 did not block all of the hyperoxia-induced capillary constriction, suggesting that a mechanism other than 20-HETE generation also contributes. We ruled out the possibility of an extra vasoconstriction arising from suppression, in hyperoxic solution, of vasodilating adenosine production in the hypoxic centre of brain slices oxygenated with 20% O_2_, or from a change of astrocyte P2X_1_ receptor activation by ATP. Blocking A1 or A2a receptors did not lead to a significant diameter change in the normoxic condition (20% O_2_), implying that a lowering of adenosine production by hyperoxia is not the cause of the hyperoxic vasoconstriction. Similarly, blocking P2X_1_ receptors had no significant effect on the capillary diameter in 20% O_2_ or on the constriction evoked by 95% O_2_, and blocking the EP4 receptor that mediates the effects of prostaglandin E_2_ on pericytes has no significant effect^44^ on the capillary diameter in 20% O_2_.

For all the experiments investigating the involvement of ROS, ET, adenosine, ATP and EP_4_ receptors, we employed concentrations of drugs that have previously been shown to block the mechanisms targeted. However, we cannot rule out the possibility that a higher concentration of the drugs used would have suppressed the hyperoxia-induced vasoconstriction.

In conclusion, hyperoxia evokes capillary constriction as a result of the higher [O_2_] promoting 20-HETE production, which evokes a [Ca^2+^]_i_ increase in pericytes, leading to pericyte contraction. Researchers should be aware that using 95% O_2_ to oxygenate brain slices (especially common in electrophysiological experiments) may cause hyperoxia and influence the outcome of the studies. Moreover, when administering O_2_, clinicians should avoid generating hyperoxia which can result in a significant reduction of cerebral blood flow that might lead to poorer brain function, especially in critically ill patients who are at risk of receiving excess oxygen.^78^ Reductions of cerebral blood flow occurring in spreading depression,^79^ stroke.^80^ cardiac arrest^81^ and subarachnoid haemorrhage^82^ have previously been shown to involve 20-HETE production. Our data add hyperoxic capillary vasoconstriction to this list.

## Supplemental Material

sj-xlsx-1-jcb-10.1177_0271678X221111598 - Supplemental material for Hyperoxia evokes pericyte-mediated capillary constriction Click here for additional data file. Supplemental material, sj-xlsx-1-jcb-10.1177_0271678X221111598 for Hyperoxia evokes pericyte-mediated capillary constriction by Chanawee Hirunpattarasilp, Anna Barkaway, Harvey Davis, Thomas Pfeiffer, Huma Sethi and David Attwell in Journal of Cerebral Blood Flow & Metabolism
